# Effect of Concomitant Administration of L-Glutamine and Cycloart-23-ene-3β, 25-diol (B2) with Sitagliptin in GLP-1 (7–36) Amide Secretion, Biochemical and Oxidative Stress in Streptozotocin - Nicotinamide Induced Diabetic Sprague Dawley Rats

**DOI:** 10.1371/journal.pone.0072817

**Published:** 2013-08-30

**Authors:** Sachin L. Badole, Swapnil M. Chaudhari, Pranita P. Bagul, Sagar P. Mahamuni, Rekha D. Khose, Anuja C. Joshi, Chandrashekhar G. Raut, Anand A. Zanwar

**Affiliations:** 1 Department of Pharmacology, PES’s Modern College of Pharmacy, Pune, India; 2 High Containment Laboratory, Microbial Containment Complex, National Institute of Virology, Pune, India; 3 Center for Innovation in Nutrition, Health, Disease, Interactive Research School for Health Affairs, Medical college campus, Bharati Vidyapeeth University, Pune, India; University of Michigan Medical School, United States of America

## Abstract

Previously we have reported that, cycloart-23-ene-3β, 25-diol (called as B2) and L-glutamine stimulated glucagon like peptide-1 (GLP-1) (7–36) amide secretion diabetic rats. The objective of present investigation was to investigate the concomitant administration of cycloart-23-ene-3β, 25-diol+sitagliptin and L-glutamine+sitagliptin in streptozotocin - nicotinamide induced diabetic Sprague Dawley. Type 2 diabetes was induced in overnight fasted male Sprague Dawley rats pre-treated with nicotinamide (100 mg/kg, i.p.) followed by administration of streptozotocin (55 mg/kg, i.p.) 20 min after. The rats were divided into; I- non-diabetic, II- diabetic control, III- Sitagliptin (5 mg/kg, p.o.)+cycloart-23-ene-3β, 25-diol (1 mg/kg, p.o.), IV- Sitagliptin (5 mg/kg, p.o.)+L-glutamine (1000 mg/kg, p.o.). The concomitant treatment of cycloart-23-ene-3β, 25-diol and L-glutamine with sitagliptin was 8 weeks. Plasma glucose, body weight, food and water intake were determined every week. Glycosylated haemoglobin, lipid profile, plasma and colonic active (GLP-1) (7–36) amide, plasma and pancreatic insulin, histology of pancreata and biomarkers of oxidative stress were measured after 8^th^ week treatment. Concomitant administration of cycloart-23-ene-3β, 25-diol and L-glutamine with sitagliptin significantly (p<0.001) reduced plasma glucose, glyoxylated haemoglobin, lipid profile and oxidative stress parameters compared to diabetic control groups. Both concomitant treatment increased plasma and pancreatic insulin as well as plasma and colonic active (GLP-1) (7–36) amide secretion. Histological analysis by Gomori staining observed less destruction of pancreatic β cells. The result obtained from this study; it is concluded that concomitant administration of cycloart-23-ene-3β, 25-diol+sitagliptin and L-glutamine+sitagliptin showed additive antihyperglycaemic effect in diabetic rats.

## Introduction

In previous study, we have reported that cycloart-23-ene-3β, 25-diol isolated from *Pongamia pinnata* showed antidiabetic activity [1] due to increased glucagon like peptide 1 (GLP-1) and insulin secretion [2] in diabetic animals. Oral L-glutamine is a non-essential amino acid which is able to decreased plasma glucose level in a dose dependent manner. It increased plasma and pancreatic insulin, plasma and colonic active GLP-1 (7–36) amide secretion as well as decreased oxidative stress in streotpzotocin - nicotinamide induced type 2 Sprague Dawley diabetic rats [3].

There is an increasing tendency towards using concomitantly drugs for the treatment of diabetes. The increased use of bioactive medicinal compounds in communities where people are also receiving approved medicines suggests that adverse herb–drug interactions may be of significant public health outcome. Biologically active constituents or amino acids interaction with conventional drugs is consequently a concern. When administered in combination with prescription medication, biologically active constituents or amino acids may positively change the pharmacokinetics [4] as well as the pharmacodynamics [5] of prescription medications. However, to date there is little evidence relating to herb–drug interaction in the case of antidiabetic medicines and the understanding of the involved mechanisms is also far from complete [6].

The interaction study of cycloart-23-ene-3β, 25-diol and L-glutamine with sitagliptin in diabetic animals or patients has not been scientifically investigated. The objective of this investigation was to investigate the concomitant administration of cycloart-23-ene-3β, 25-diol+sitagliptin and L-glutamine+sitagliptin in streptozotocin - nicotinamide induced diabetic Sprague Dawley (SD) rats.

## Materials and Methods

### Drugs and Chemicals

Streptozotocin, nicotinamide and L- glutamine were purchased from Sigma chemical co. USA. Glucose oxidase peroxidase (GOD/POD) kit (Acuurex, India), tween-80 (Research-Lab, India), (Sigma chemical Co. USA) and sitagliptin (Januvia®^,^ Merck and CO., INC., USA) were purchased from individual vendors.

### Animals and Research Protocol Approval

Male SD rats (180–220 g) were procured in PES’s Modern College of Pharmacy, Pune, India and housed in an air-conditioned room at a temperature of 25±2°C and relative humidity of 45 to 55% under 12-h light: 12-h dark cycle. The animals had free access to food pellets (Chakan Oil Mills, Pune, India) and water was provided *ad libitum*. The experimental protocol was approved by the Institutional Animal Ethics Committee (IAEC) constituent under the PES’s Modern College of Pharmacy, Pune, India (IAEC protocol no MCP/IAEC/23/2011).

### Preparation of Drugs Solution

Cycloart-23-ene-3β, 25-diol was emulsified with 2% tween-80. L-glutamine and sitagliptin were dissolved in distilled water. Streptozotocin was dissolved in citrate buffer (pH 4.5) and nicotinamide in normal physiological saline.

### Induction of Diabetes

Diabetes was induced in overnight fasted Sprague Dawely rats by using intraperitonial injection of streptozotocin (55 mg/kg). Nicotinamide (100 mg/kg, i.p.) was administered 20 min before streptozotocin [2],[3],[7]. Non-diabetic rats were administered physiological saline. On day 15 the plasma glucose was measured by glucose oxidase peroxidase (GOD/POD) method. The animals having plasma glucose level above 200 mg/dl were labeled as diabetic.

### Plasma Glucose Level

The rats were divided into following groups (n = 6) *viz*;

- I: non-diabetic,
- II: diabetic control,
- III: cycloart-23-ene-3β, 25-diol (1 mg/kg)+sitagliptin (5 mg/kg),
- IV: L-glutamine (1000 mg/kg)+sitagliptin (5 mg/kg).

All groups were diabetic apart from group I. Sitagliptin+cycloart-23-ene-3β, 25-diol, and sitagliptin+L-glutamine were administered orally concomitantly within a 20 min interval. All drugs concomitantly administered once a day at pre-determined time for 8 weeks. Plasma glucose was determined by using GOD/POD method each week (0 to the 8^th^ week).3

### Body Weight, Food and Water Intake

Body weight, food and water intake of each rats were recorded daily but data was accessible only at day 0 (15 days after injection of streptozotocin–nicotinamide) and at each consequent weeks [2], [3].

### Glycosylated Haemoglobin and Lipid Profile

At the end of study, glycosylated haemoglobin was determined by Nycocard reader (Axis shield, Norway). Cholesterol, triglycerides, high density lipoproteins and low density lipoprotein levels were determined by specific kits (Microlab 300, Merck, Netherland).

### Active GLP-1 (7–36) Amide Concentration in Plasma and Rat Colon

Active GLP-1 (7–36) amide concentrations in plasma and insulin level were determined in blood as well as colon tissue by using previous reported method [2], [3].

After 8 weeks of treatment, food was withheld and 8 h later rats were anaesthetized. Portal vein blood samples were collected in EDTA tubes containing dipeptidyl peptidase IV inhibitor (Millipore, USA). Blood samples were centrifuged (20 min at 804 g at 4°C temperature) and plasma samples were separated and stored at −80 0C. Active GLP-1 (7–36) amide was measured by using GLP-1 Active ELISA kit (Millipore, USA) and ELISA reader (FluoStar Omega BMG Lab tech, Germany).

### Colon Samples

At the end of study, rats were sacrifice by cervical dislocation, colon was instantly excised, flushed with ice-cold saline buffer and divided into 2 cm segments that are just after the caecal junction, the middle of the colon and just before the rectum. Intestinal segments (n = 6) were carried out for determination of active GLP-1 (7–36) amide by previously reported method. [2], [3] Colon was extracted with ethanol-acid solution (100% ethanol:sterile water:12M HCl, 74∶25∶1 v/v/v) using 5 ml/g tissue. Samples were homogenized and placed for 24 h at 4°C. Homogenates were centrifuged (20 min at 2465 g) and supernatant was decanted and diluted 500 fold in physiological saline solution. Concentrations of colon active GLP-1 (7–36) amide were measured by using above mentioned plasma GLP-1 (7–36) amide analysis.

### Plasma and Pancreatic Insulin

Plasma insulin was assayed by Accubind, ELISA Micorwell Insulin kit (USA) as per manufacturer instruction.

Pancreatic insulin levels were measured by previously reported method. [1]–[3] Pancreas were homogenized in an ice cold concentrated hydrochloric acid:ethanol (1∶4, v/v) and centrifuged (5204 g at 4°C). The subsequent supernatant was pooled and stored in amber color vials at −80°C until assayed.

### Histopathology of Pancreata by Gomori Staining

Small piece of pancreas was cut by scissor and stored in 10% neutral formalin solution. Pancreatic tissue was processed for Gomori staining in order to assess the morphology of pancreatic β cells [8]. The photomicrographs of each tissue section were observed using Cell imaging software for Life Science microscopy (Olympus soft imaging solution GmbH, Munster, Germany).

### Enzymatic Biomarkers of Oxidative Stress

Liver was isolated from all rats and were cut into small pieces, placed in chilled 0.25 M sucrose solution and blotted on a filter paper. The tissues were then homogenized in 10% chilled tris hydrochloride buffer (10 mM, pH 7.4) by tissue homogenizer (Remi Motors, Mumbai, India) and centrifuged at 12000 r.p.m. for 15 min at 0°C using Eppendorf 5810-R high speed cooling centrifuge [1]–[3].

The biomarkers of oxidative stress were selected *viz*; malondialdehyde, reduced glutathione, superoxidase dismutase, glutathione peroxidase, glutathione S transferase and total protein. Malondialdehyde content in supernatant of rat liver was determined by previously reported method [9]. The assay of reduced glutathione was carried out by method of Moron et al [10]. The superoxidase dismutase activity was determined by the method of Misera and Fridovich [11]. Glutathione S transferase and glutathione peroxidase were determined by the method of Habig et al., [12] and Rotruck et al., [13] respectively. Protein concentration was determined by using method Lowry et al. [14].

### Statistical Analysis

Data was expressed as mean ±S.E.M. Statistical analysis was carried out by one way ANOVA followed by post hoc Tukey test performed using GraphPad InStat version 3.00 for Windows VistaTM BASIC, GraphPad Software, San Diego, California, USA. *p*<0.05 was considered statistically significant.

## Results

### Plasma Glucose Level

**Figure 1:**
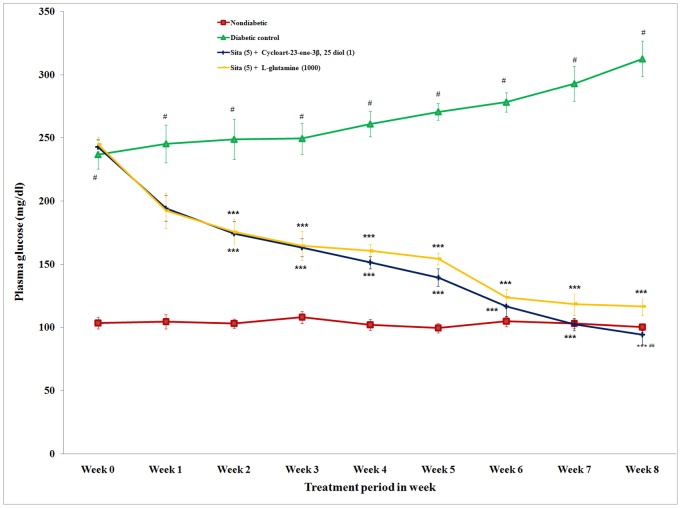
Effect of concomitant administration of cycloart-23-ene-3β, 25-diol and L-glutamine with sitagliptin on plasma glucose level in streptozotocin-nicotinamided induced diabetic rats. Values are mean ± S.E.M., n = 6 in each group; statistical analysis by one way ANOVA followed by Post hoc Tukey’s test using Graphpad Instat software; *p* value^ #^<0.001 compared to non-diabetic groups; *<0.05; **<0.01; ***<0.001 compared to diabetic control group; ^##^ <0.01 compared to L-glutamine (1000 mg/kg) + Sita (5 mg/kg).

The actual values of plasma glucose are presented in Fig. 1. The difference in plasma glucose level before (day 0) and after (week 8) drug treatment was calculated. Repeated concomitant administration (once a day for 8 week) of cycloart-23-ene-3β, 25-diol and L-glutamine with sitagliptin caused a significant (p<0.001) reduction in the plasma glucose compared to the diabetic group. The reduction in the plasma glucose level of concomitant administrations of cycloart-23-ene-3β, 25-diol and L-glutamine with sitagliptin was 157.55 and 128.17 mg/dl, respectively. The concomitant administration of cycloart-23-ene-3β, 25-diol+sitagliptin and L-glutamine+sitagliptin showed an antihyperglycaemic effect in diabetic rats (Fig. 1). Concomitant administration of cycloart-23-ene-3β, 25-diol+sitagliptin showed significant (p<0.01) decrease in plasma glucose level as compared to L-glutamine+sitagliptin.

### Body Weight, Food and Water Intake

The initial body weights were similar in all groups. Body weight of diabetic control significantly (p<0.001) decreased during study period compared to nondiabetic group. However, diabetic rats concomitantly treated cycloart-23-ene-3β, 25-diol+sitagliptin and L-glutamine+sitagliptin significantly (p<0.001) prevented a decrease in the body weight (Table 1).

**Table 1:** Effect of concomitant administration of cycloart-23-ene-3β, 25 diol and L-glutamine with sitagliptin on body weight, food and water intake in streptozotocin – nicotinamide induced diabetic rats.

Parameters		Non-diabetic	Diabetic control	Cycloart-23-ene-3β, 25 diol(1 mg/kg) + Sitagliptin (5 mg/kg)	L-glutamine (1000 mg/kg)+Sita (5 mg/kg)	
Body weight (g/day)	0	198.83±5.83	208.20±6.82	208.74±2.32	201.32±2.43	
	Week 1	206.83±4.57	205.00±6.73	209.19±3.65	202.87±3.76	
	Week 2	213.50±5.73	196.40±6.97	205.32±4.19	200.62±6.18	
	Week 3	223.83±5.9	190.20±9.02^#^	213.31±5.38*	197.75±3.38	
	Week 4	236.66±7.62	174.40±5.77^#^	212.96±6.27**	198.00±5.42	
	Week 5	245.00±7.60	169.00±4.78^#^	215.02±5.38**	197.14±2.63*	
	Week 6	252.33±7.27	169.20±3.99^#^	218.59±6.12***	197.50±4.74*	
	Week 7	265.33±8.27	163.00±3.46^#^	219.63±4.91***	196.83±6.43**	
	Week 8	284.50±5.78	160.00±3.11^#^	225.46±6.23***	195.49±6.40***	
Food intake (g/day)	0	16.78±0.69	26.15±0.55^#^	26.37±0.64	26.43±0.53	
	Week 1	18.31±0.57	26.66±0.42^#^	25.03±0.53	26.19±0.69	
	Week 2	18.48±0.58	28.16±0.70^#^	22.32±0.79	25.65±0.50	
	Week 3	19.84±0.97	30.50±0.62^#^	22.09±0.71	25.13±0.81***	
	Week 4	19.34±0.34	33.66±0.75^#^	23.93±0.83	24.07±0.71***	
	Week 5	19.68±0.36	34.37±0.42^#^	23.18±0.61	24.09±0.69***	
	Week 6	19.80±0.76	38.13±0.68^#^	23.73±0.79	23.49±0.30***	
	Week 7	18.50±0.64	37.16±0.60^#^	22.07±0.68	23.45±0.47***	
	Week 8	19.80±0.44	39.08±0.73^#^	21.17±0.49	23.05±0.61***	
Water Intake ml/day)	0	21.83±0.60	69.50±2.33^#^	72.07±2.27	71.73±1.82	
	Week 1	22.50±0.49	69.83±1.95^#^	65.71±2.43	69.54±2.19	
	Week 2	22.61±0.51	71.00±1.75^#^	53.79±3.18	61.16±4.19**	
	Week 3	22.83±0.70	79.66±2.33^#^	47.31±2.69	49.50±3.14***	
	Week 4	22.33±0.61	82.33±2.37^#^	34.09±2.91	41.71±3.11***	
	Week 5	22.05±0.53	90.00±3.58^#^	28.93±1.79	32.20±2.38***	
	Week 6	21.16±0.47	103.33±2.45^#^	22.86±1.95	26.16±3.13***	
	Week 7	22.24±0.3	108.43±3.71^#^	22.48±2.39	25.09±2.17***	
	Week 8	22.37±0.58	116.43±2.04^#^	21.91±2.04	23.74±1.06***	

Values are mean ± S.E.M., n = 6 in each group; statistical analysis by one way ANOVA followed by Post hoc Tukey’s test using Graphpad Instat software; p value^ #^<0.001 compared to non-diabetic groups;

* <0.05;

** <0.01;

*** <0.001 compared to diabetic control group.

Food and water intake were significantly (p<0.001) reduced in concomitantly treated groups compared to diabetic control group (Table 1).

### Glycosylated Haemoglobin and Lipid Profile

Concomitantly administration of cycloart-23-ene-3β, 25-diol+sitagliptin and L-glutamine+sitagliptin significantly (p<0.001) lower glycosylated haemoglobin compared to diabetic control group. However concomitant administration of cycloart-23-ene-3β, 25-diol+sitagliptin showed non-significant lower in glycosylated haemoglobin level as compared to L-glutamine+sitagliptin (Table 2).

**Table 2:** Effect of concomitant administration of cycloart-23-ene-3β, 25 diol and L-glutamine with sitagliptin on glycosylated hemoglobin, lipid profile and antioxidant parameters in streptozotocin – nicotinamide induced diabetic rats.

Parameters		Non-diabetic	Diabetic Control	Cycloart-23-ene-3β, 25 diol(1 mg/kg)+Sita (5)	L-glutamine (1000 mg/kg)+Sita (5)	
	Glycosylated haemoglobin (%)	7.48±0.13	13.02±0.47^#^	6.98±0.71***	7.37±0.91***	
**Lipid Profile**	Cholesterol (mg%)	41.33±0.95	91.00±1.88^#^	45.09±2.90***	49.43±1.76***	
	Triglycerides (mg%)	62.83±3.16	176.16±2.21^#^	71.87±2.38***	82.08±3.17***	
	High density lipoprotein (mg%)	15.33±0.84	7.16±0.60^#^	14.24±1.13***	14.01±0.32***	
	Very low density lipoprotein (mg%)	12.57±0.63	35.23±0.44^#^	16.42±0.78***	17.04±0.73***	
	Low density lipoprotein (mg%)	13.43±1.50	48.60±1.79^#^	14.03±3.0***	14.86±2.62***	
**Antioxidant parameters**	Malonaldehyde (nM of MDA/mg protein)	2.89±0.084	5.79±0.23^#^	2.56±0.21***	2.97±0.24***	
	Reduced glutathione (µg of GSH/mg protein)	29.06±1.29	17.71±0.52^#^	34.06±0.94***,##	25.43±1.29***	
	Superoxide dismutase (U/mg protien)	15.41±1.2	7.37±0.89^#^	16.86±1.76***	15.62±1.91**	
	Glutathione peroxidase (µmole)	31.76±0.58	21.56±0.83^#^	33.76±0.87***,##	29.03±0.83*	
	Glutathione S transferase (µmole)	133.4±8.04	87.43±6.79^#^	136.41±4.36***	132.32±5.19***	

Values are mean ± S.E.M., n = 6 in each group; statistical analysis by one way ANOVA followed by Post hoc Tukey’s test using Graphpad Instat software; *p* value^ #^<0.001 compared to non-diabetic groups;

* <0.05;

** <0.01;

*** <0.001 compared to diabetic control group;

## <0.01 compared to L-glutamine (1000 mg/kg)+Sita (5 mg/kg).

Serum cholesterol, triglycerides, very low density lipoprotein and low density lipoprotein and concomitantly treated groups were significantly (p<0.001) decreased. High density lipoprotein levels significantly (p<0.001) increased in all treated groups compared to diabetic control group. However concomitant administration of cycloart-23-ene-3β, 25-diol+sitagliptin showed non-significant decrease in serum cholesterol, triglycerides, very low density lipoprotein and low density lipoprotein level and non-significant incrase in high density lipoprotein levels as compared to L-glutamine+sitagliptin (Table 2).

The concomitant administration of cycloart-23-ene-3β, 25-diol+sitagliptin and L-glutamine+sitagliptin treatment showed a reduction in glycosylated haemoglobin as well as lipid profile in diabetic rats.

### Active GLP-1 (7–36) Amide Concentration in Plasma and Rat Colon

**Figure 2:**
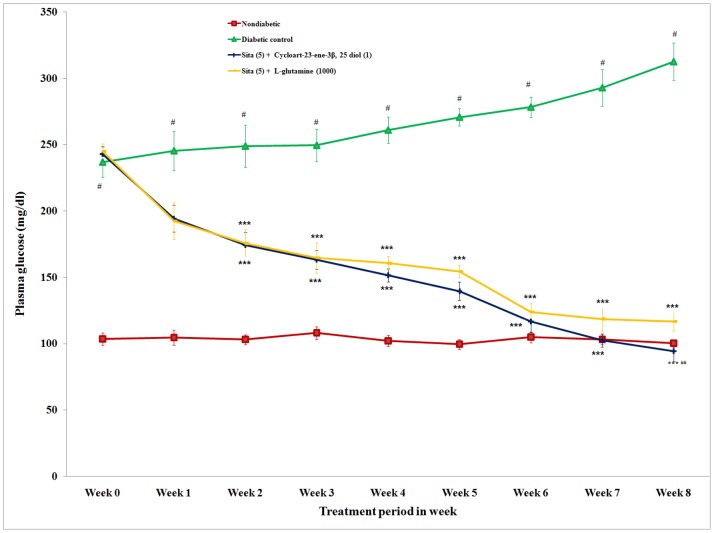
Effect of concomitant administration of cycloart-23-ene-3β, 25-diol and L-glutamine with sitagliptin on active GLP-1 (7–36) amide concentrations in plasma. Values are mean ± S.E.M., n = 6 in each group; statistical analysis by one way- ANOVA followed by Post hoc Tukey’s test using Graphpad Instat software; *p* value^ #^<0.001 compared to non-diabetic groups; *<0.05; **<0.01; ***<0.001 compared to diabetic control group.

The plasma concentration of active GLP-1 (7–36) amide in non-diabetic group was 3.80±0.30 pM. Active GLP-1 (7–36) amide concentrations in plasma were significantly (p<0.001) increased in concomitant administration of cycloart-23-ene-3β, 25-diol+sitagliptin (7.14±0.32 pM) as well as L-glutamine+sitagliptin (6.43±0.27 pM) treated diabetic rats than diabetic control rats (2.65±0.13 pM). However concomitant administration of cycloart-23-ene-3β, 25-diol+ sitagliptin showed non-significant increase in plasma concentration of active GLP-1 (7–36) amide as compared to L-glutamine+sitagliptin (**Fig. 2**).

**Figure 3:**
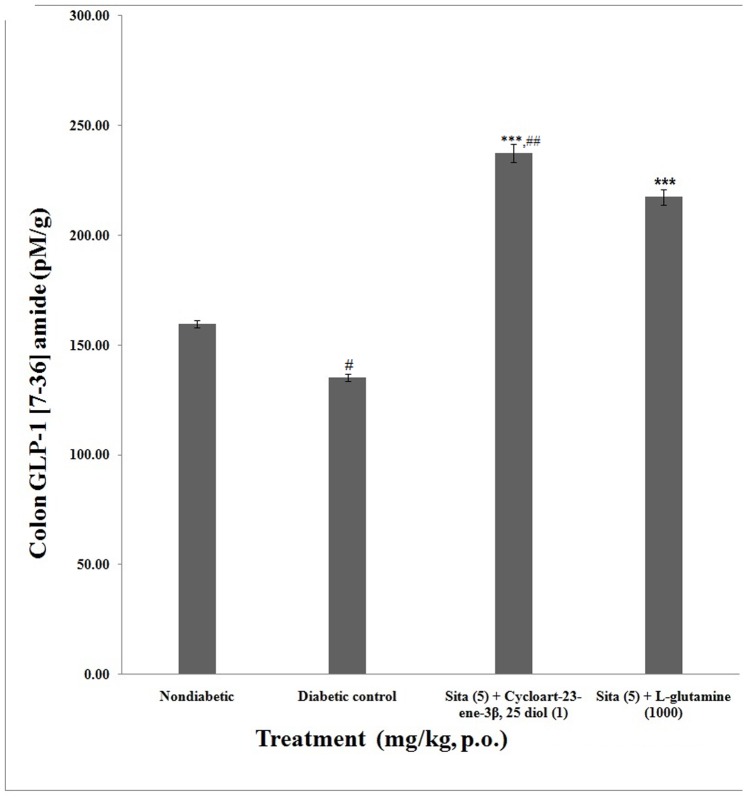
Effect of concomitant administration of cycloart-23-ene-3β, 25-diol and L-glutamine with sitagliptin on active GLP-1 (7–36) amide concentrations in rat colon. Values are mean ± S.E.M., n = 6 in each group; statistical analysis by one way- ANOVA followed by Post hoc Tukey’s test using Graphpad Instat software; *p* value^ #^<0.001 compared to non-diabetic groups; *<0.05; **<0.01; ***<0.001 compared to diabetic control group, ^##^ <0.01 compared to L-glutamine (1000 mg/kg) + Sita (5 mg/kg).

The rat colon concentration of active GLP-1 (7–36) amide in non-diabetic group was found to be 159.50±1.69 pM/g. Colon concentration in active GLP-1 (7–36) amide were significantly (p<0.001) higher in concomitant administration of cycloart-23-ene-3β, 25-diol+sitagliptin (237.29**±**4.03 pM/g) as well as L-glutamine+sitagliptin (217.42±3.49 pM) treated animals than diabetic rats (135.17±1.70 pM/g). Concomitant administration of cycloart-23-ene-3β, 25-diol+ sitagliptin showed significant (p<0.01) increase in colonic concentration of active GLP-1 (7–36) amide as compared to L-glutamine+sitagliptin (**Fig. 3**).

The concomitant administration of cycloart-23-ene-3β, 25-diol+sitagliptin and L-glutamine+sitagliptin showed an increased in plasma as well as colonic concentration of active GLP-1 (7–36) amide.

### Plasma and Pancreatic Insulin

**Figure 4:**
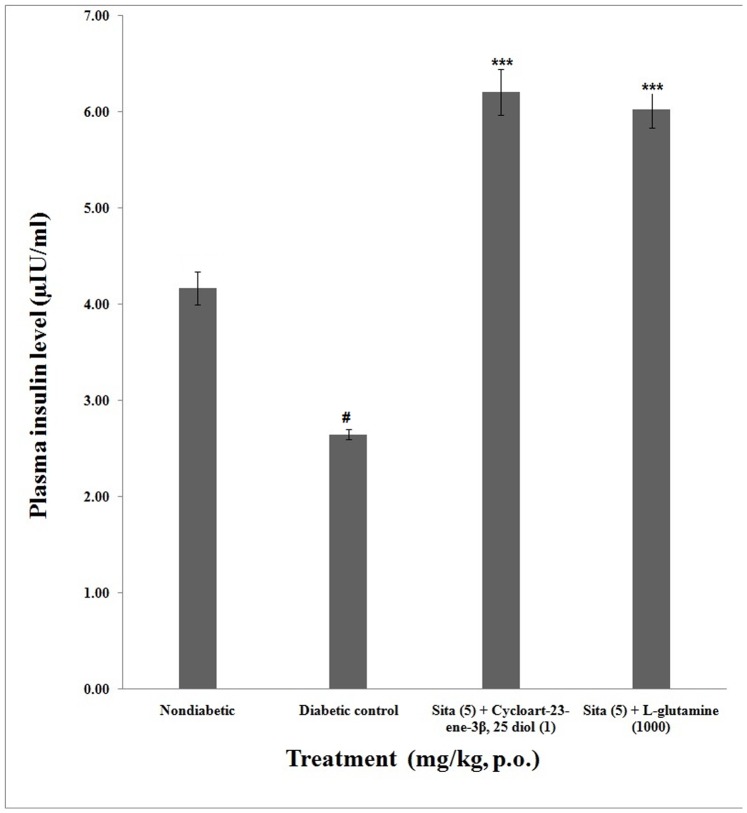
Effect of concomitant administration of cycloart-23-ene-3β, 25-diol and L-glutamine with sitagliptin on plasma insulin. Values are mean ± S.E.M., n = 6 in each group; statistical analysis by one way- ANOVA followed by Post hoc Tukey’s test using Graphpad Instat software; *p* value^ #^<0.001 compared to non-diabetic groups; *<0.05; **<0.01; ***<0.001 compared to diabetic control group.

The plasma insulin level of non-diabetic group was 4.17±0.16 µIU/ml. The plasma insulin level of concomitant administration of cycloart-23-ene-3β, 25-diol+sitagliptin (6.21**±**0.24 µIU/ml) as well as L-glutamine+sitagliptin (6.03±0.91 µIU/ml) were significantly (p<0.001) increased compared to diabetic control (0.57±0.05 µIU/ml). However concomitant administration of cycloart-23-ene-3β, 25-diol+sitagliptin showed non-significant (p<0.01) increase in plasma insulin level as compared to L-glutamine+sitagliptin (**Fig. 4**).

**Figure 5:**
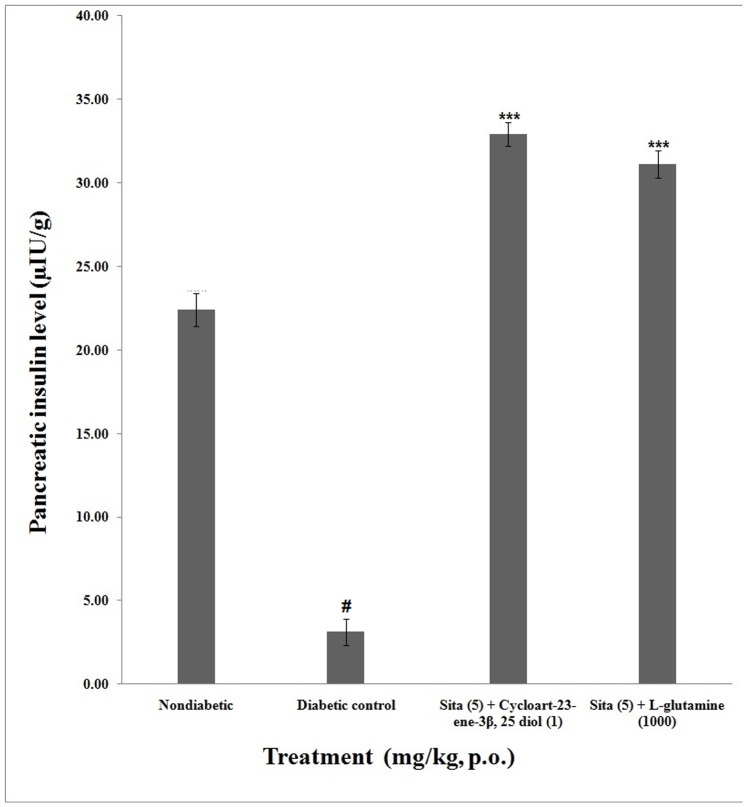
Effect of concomitant administration of cycloart-23-ene-3β, 25-diol and L-glutamine with sitagliptin on pancreatic insulin. Values are mean ± S.E.M., n = 6 in each group; statistical analysis by one way- ANOVA followed by Post hoc Tukey’s test using Graphpad Instat software; *p* value^ #^<0.001 compared to non-diabetic groups; *<0.05; **<0.01; ***<0.001 compared to diabetic control group.

The pancreatic insulin level of non-diabetic group was 22.40±0.98 µIU/g after 8 week. The pancreatic insulin level of concomitant administration of cycloart-23-ene-3β, 25-diol+sitagliptin (32.93**±**0.69 µIU/ml) as well as L-glutamine+sitagliptin (31.12±0.82 µIU/ml) were significantly (*P*<0.001) increased compared to diabetic control (6.03±0.91 µIU/g). However concomitant administration of cycloart-23-ene-3β, 25-diol+sitagliptin showed non-significant (p<0.01) increase in plasma insulin level as compared to L-glutamine+sitagliptin (**Fig. 5**).

The concomitant administration of cycloart-23-ene-3β, 25-diol+sitagliptin and L-glutamine+sitagliptin showed an increased in plasma as well as pancreatic insulin in diabetic rats.

### Histopathology of Pancreata by Gomori Staining

**Figure 6:**
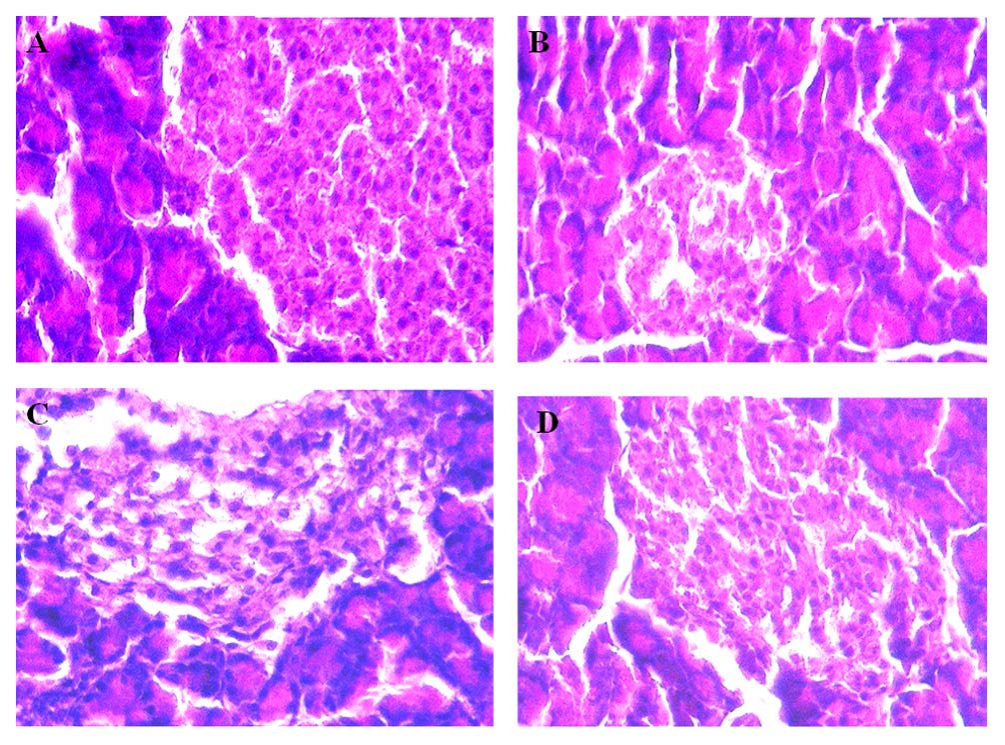
Photomicrographs of histological changes of rat pancreata (Gomori stain). A) Non-diabetic: Normal histological structure of rat pancreata showing average sized islets (red arrow) and normal sized β cells. B) Diabetic control rat pancreata showing β cells slightly elongated with more destruction (++++). C) Concomitant administration of sitagliptin (5 mg/kg, p.o.) with cycloart-23-ene-3β, 25-diol (1 mg/kg, p.o.) treated rat pancreata showing slightly elongated of the β cells with less destruction (+). D) Concomitant administration of sitagliptin (5 mg/kg, p.o.) with L-glutamine (1000 mg/kg, p.o.) treated rat pancreata showing slightly elongated of the β cells with less destruction (+). ***Grade:*** – No injury; Grade: ++++ severe injury; Grade: ++ mild injury; Grade: + Very mild injury.

Histological analysis by Gomori staining of non-diabetic SD rat pancreata showed normal histological structure, depict average sized islets and normal sized β cells (**Fig. 6A**). Diabetic control rat pancreata showed β cells slightly elongated with more destruction (Grade ++++) (**Fig. 6B**). Concomitant administration of cycloart-23-ene-3β, 25-diol+sitagliptin (**Fig. 6C**) as well as L-glutamine + sitagliptin (**Fig. 6D**) caused β cells slightly elongated with less destruction (Grade +).

### Enzymatic Biomarkers of Oxidative Stress

We observed, malondialdehyde (MDA) level was significantly (p<0.001) increased in diabetic control group compared to non diabetic group. The activities of protective antioxidant enzymes level of reduced glutathione (GSH), superoxidase dismutase (p<0.001), glutathione peroxidase (p<0.001) and glutathione S transferase (p<0.001) were significantly reduced in diabetic control group as compared with non diabetic group.

Treatment of concomitant administered drugs significantly (p<0.001) restored the contents of malondialdehyde (*p*<0.001), reduced glutathione (p<0.001), superoxidase dismutase (p<0.001), glutathione peroxidase (p<0.001) and glutathione S transferase (p<0.001) in liver homogenate as compared with diabetic group. Concomitant administration of cycloart-23-ene-3β, 25-diol+ sitagliptin showed significant (p<0.01) increase in reduced glutathione and glutathione peroxidase as compared to L-glutamine+sitagliptin.

These data indicated that concomitant administration of cycloart-23-ene-3β, 25-diol with sitagliptin treatment showed additive improved the levels of endogenous antioxidant enzymes in liver than the concomitant administration of L-glutamine with sitagliptin (**Table 2**).

## Discussion

In the present study, potent diabetogenic action of streptozotocin produced injury of the pancreatic β-cells. Nicotianmide (pyridine-3-carboxamide) is the amide form of vitamin B3 (niacin) [15]. Administration of streptozotocin showed diabetic syndrome in rats and partially protected with suitable dosages of nicotinamide is characterized by stable moderate hyperglycaemia and glucose intolerance [7].

In previous study, we reported that, administration of cycloart-23-ene-3β, 25-diol (1 mg/kg) and sitagliptin (5 mg/kg) alone reduced plasma glucose level in streptozotocin–nicotiamide induced type 2 diabetes mellitus in Sprague Dawley rats after 8 week treatment period. Cycloart-23-ene-3β, 25-diol and sitagliptin reduced lipid absorption due to increased concentration of GLP-1. Cycloart-23-ene-3β, 25-diol (1 mg/kg) and sitagliptin (5 mg/kg) treated animals showed significant increase in active GLP-1 (7–36) amide concentrations in plasma and rat colon. Proglucagon acts as precursor of GLP-1, the GLP-1 were identified after the cloning of the cDNAs and genes encoding the human proglucagon. This proglucagon gene is expressed in L-cells of intestine, ileum and colon. Activation of GLP-1 receptors on β –cells leads to rapid increased in levels of cAMP or activation of either protein kinase A or cAMP regulated guanine nucleotide exchange factor II alters ion channel activity, handling of intracellular calcium and enhances the exocytosis of insulin containing granules. There is association between increased gut proglucagon expression and enhanced intestinal levels of GLP-1. In our previous study, the levels of proglucagon mRNA expression in sitagliptin (5 mg/kg) and cycloart-23-ene-3b, 25-diol (1 mg/kg) treated groups were found significantly increased [2].

Recently we reported that, L-glutamine bind to the GLP-1 receptor in docking study. Oral administration of L-glutamine (250, 500 or 1000 mg/kg) reduced plasma glucose level in dose depended manner. The decreased plasma glucose levels as well as increased plasma and pancreatic insulin observed in previous study indicated that L-glutamine stimulated insulin secretion *via* GLP-1 stimulation in a dose depended manner. In oral glucose tolerance test, administration of L-glutamine and sitagliptin effectively prevented the increase in serum glucose and decrease insulin level without causing a hypoglycemic state as well as decreased oxidative stress. Maximum antidiabetic activity was found to be 1000 mg/kg of L-glutamine [3].

This study was aimed to determine whether sitagliptin concomitantly administered with cycloart-23-ene-3β, 25-diol or L-glutamine could increased antihyperglycemic effect or not? We observed that cycloart-23-ene-3β, 25-diol concomitantly administered with sitagliptin produced additively decreased plasma glucose levels, increased glucagon like peptide-1, insulin secretion than the concomitantly administered with L-glutamine *in vivo* model of streptozotocin- nicotinamide induced diabetes in rats.

Sitagliptin is a selective inhibitor of the enzyme dipeptidyl peptidase-4 (DPP-4), which metabolized the naturally occurring incretin hormones GLP-1 resulting in enhanced glucose-dependent insulin secretion from the pancreas and decreased hepatic glucose production [16].

The concomitant administration of cycloart-23-ene-3β, 25 or L-glutamine with sitagliptin was effective in reducing the plasma glucose level after 1 week of treatment and thereafter. Concomitant administration of cycloart-23-ene-3β, 25-diol+sitagliptin and L-glutamine+sitagliptin increased GLP-1 secretion in both rat plasma as well as colon. Moreover more increased in GLP-1 found in concomitant administered in cycloart-23-ene-3β, 25-diol with sitagliptin than the concomitantly administered with L- glutamine with sitagliptin treatment. In previous molecular docking study suggested that, there is no remarkable difference in the binding modes of sitagliptin, cycloart-23- ene-3b, 25-diol and L-glutamine. Sitagliptin (−8.25) [2] and cycloart-23-ene-3β, 25-diol (−7.87) [2] portrayed better GLP-1 receptor agonistic activity compared with L-glutamine (−6.87) [3].

DPP-4 inhibitors do not affect the body weight [17]. Concomitant administration of cycloart-23-ene-3β, 25-diol or L-glutamine with sitagliptin protected against weight loss seems to be due to their ability to reduce hyperglycaemia. Similar to characteristics of type 2 diabetes in humans; polyphagia and polydipsia were observe in diabetic control rats, whereas no marked fluctuations were observe in food and water intake of non-diabetic rats. Concomitantly treated rats showed significant reduction in food and water intake compared to diabetic control.

Sitagliptin reduced glycosylated hemoglobin, fasting and postprandial glucose by glucose-dependent stimulation of insulin secretion [18] Cycloart-23-ene-3β, 25-diol [1], [2] and L-glutamine [3] reduced glycosylated hemoglobin in diabetic animals. Similar results observed in present study. We observed, concomitant administration of cycloart-23-ene-3β, 25-diol+sitagliptin and L-glutamine+sitagliptin produced more reduction in glycosylated hemoglobin comparable to the isolated administration of cycloart-23-ene-3β, 25-diol [2] and L-glutamine [3].

Diabetes mellitus is often linked with abnormal lipid metabolism [19] which is contribute in metabolic disorder in diabetic complications [20]. Hyperglycaemia produced noticeable increased in serum triglycerides and total cholesterol [21]. This hyperlipidemia related with diabetes mellitus may be attributed to lack of insulin [22]. Normalization of the plasma glucose resulted in significant reductions in serum cholesterol, triglycerides and protein [23]. GLP-1 reduces lipid absorption in rats [24], [25]. In the present study, prominent serum total cholesterol, triglycerides and decreased high density lipoprotein were observed in diabetic control. Concomitant administration of cycloart-23-ene-3β, 25-diol or L-glutamine with sitagliptin reduced lipid absorption than isolated treatment of cycloart-23-ene-3β, 25-diol [2] and L-glutamine [3].

Streptozotocin produced effect on glucose and insulin homeostasis replicate the toxin-induced abnormality in β cell function [26]. The decreased plasma glucose level as well as increased plasma and pancreatic insulin observed in our study indicate that concomitant treatment enthused insulin secretion via GLP-1 stimulation [2], [3]. This assumption is further supported by the pancreatic histology which showed protection of pancreatic β cells from toxic effect of streptozotocin. Concomitant treated animals showed less destruction of pancreatic β cells.

Increased oxidative stress is a widely accepted participant in the development and progression of diabetes and its complications [27]. Both experimental and clinical studies suggested that, there are dose links among hyperglycemia, oxidative stress and diabetic complications [28]. In the present study, we measured activities of antioxidant enzymes in liver *viz*; malondialdehyde, reduced glutathione, superoxidase dismutase, glutathione peroxidase and glutathione S transferase. The level of malondialdehyde (MDA) increased and level of reduced glutathione (GSH) was reduced in diabetic control group. While, treatment groups restore the content of malondialdehyde and reduced glutathione. The defensive enzymes superoxidase dismutase, glutathione peroxidase, glutathione S transferase were found to be reduced in diabetic control animals. The activities of the superoxidase dismutase, glutathione peroxidase, glutathione S transferase were significantly increased in concomitant treatment.

It is concluded that, concomitant administration of cycloart-23-ene-3β, 25-diol+sitagliptin and L-glutamine+sitagliptin showed additive antihyperglycaemic effect in diabetic rats.
